# Latitude and Altitude Influence Secondary Metabolite Production in Peripheral Alpine Populations of the Mediterranean Species *Lavandula angustifolia* Mill.

**DOI:** 10.3389/fpls.2018.00983

**Published:** 2018-07-05

**Authors:** Sonia Demasi, Matteo Caser, Michele Lonati, Pier L. Cioni, Luisa Pistelli, Basma Najar, Valentina Scariot

**Affiliations:** ^1^Department of Agricultural, Forest and Food Sciences, University of Torino, Grugliasco, Italy; ^2^Department of Pharmacy, University of Pisa, Pisa, Italy

**Keywords:** lavender, Lamiaceae, volatile organic compounds, essential oils, ecological gradient

## Abstract

*Lavandula angustifolia* Mill. has a great economic importance in perfumery, cosmetics, food manufacturing, aromatherapy, and pharmaceutical industry. This species finds its phytosociological optimum in the sub-Mediterranean region. Latitudinal and altitudinal gradients are expected to affect species diversification in peripheral alpine populations. In this study, phenotypic traits including morphometric parameters, volatile organic compounds (VOCs) and essential oils (EOs) were analyzed in lavender peripheral populations selected in order to explore different ecological conditions. Plants were cultivated under uniform conditions to observe variations due to the genetic adaptation to native environments and to exclude the short-term response to environmental factors. Results showed qualitatively and quantitatively intra-specific variations in secondary metabolites, mainly along the latitudinal gradient, while minor effect was attributable to the altitude. This latter affected more the morphometric parameters. As the latitude augmented, VOCs showed lower content of monoterpene hydrocarbon (mh) and higher content of oxygenated monoterpenes (om); whereas EOs showed higher content of mh and non-terpene derivatives (nt) and lower content of sesquiterpene hydrocarbons (sh). Lavender aroma and EO composition varied in every population, for a total of 88 and 104 compounds identified, respectively. Eleven and 13 compounds were responsible for 95% of the dissimilarity, with linalool, linalyl acetate and 1,8-cineole as major contributors. As the latitude augmented, linalool decreased and 1,8-cineole increased while linalyl acetate content was unaffected. These results are discussed with regards to the potential adoption of the lavender peripheral alpine populations for the improvement of quality and productivity of lavender cultivations, especially in mountainous areas.

## Introduction

The genus *Lavandula* (Lamiaceae family) comprises approximately 39 species, with a natural occurrence in the Mediterranean region, to the Arabian Peninsula, South West Asia and India (Lis-Balchin, 2003). This genus is constituted by small evergreen shrubs, with aromatic foliage and flowers, and due to its great economic importance this genus was the subject of several studies. Indeed, 1,000 tons of lavandin essential oil (*L*. × *intermedia* Emeric ex Loisel., EO) are produced worldwide every year, while only 200 tons of EO from lavender (*L. angustifolia* Mill.) together with 200 tons of EO from spike lavender (*L. latifolia* Medik.) are produced (Lesage-Meessen et al., 2015). Lavender dried flowers and EOs are commonly employed in perfumery and cosmetics, food manufacturing and aromatherapy (Cavanagh and Wilkinson, 2002; Gonçalves and Romano, 2013; Prusinowska and Smigielski, 2014). Biological and antioxidant properties of lavender EO have been extensively assessed (Cavanagh and Wilkinson, 2002; Raut and Karuppayil, 2014; Shahdadi et al., 2017). Numerous therapeutic activities have been reported, such as convulsion relief, anxiety and depression improvement, along with a positive effect to treat several neurological disorders (Cavanagh and Wilkinson, 2002; Caputo et al., 2016; López et al., 2017; Rahmati et al., 2017). The highest quality lavender oil comes from *L. angustifolia* (syn. *L. spica* L., *L. officinalis* Chaix., *L. vera* DC.) inflorescences, which contain high levels of linalyl acetate and linalool and low amount of camphor, considered positive features by both cosmetic and pharmaceutical industry (Cavanagh and Wilkinson, 2002; Kim and Lee, 2002; Shellie et al., 2002). Although the genus *Lavandula* has been widely studied in wild and controlled environment (Da Porto and Decorti, 2008; Da Porto et al., 2009; González-Coloma et al., 2011; Gonçalves and Romano, 2013; Pistelli et al., 2013, 2017; Hassiotis et al., 2014; Lesage-Meessen et al., 2015; Caputo et al., 2016; Kirimer et al., 2017; López et al., 2017; Rahmati et al., 2017), lavender aroma profile and its EO composition are continuously subjected to many investigations (Prusinowska and Smigielski, 2014).

Secondary metabolites from plants are involved in several functions such as attraction for pollinators, signaling between plants, defense and protection against biotic and abiotic stresses (Knudsen et al., 2006; Bakkali et al., 2008; Dudareva et al., 2013; Raut and Karuppayil, 2014; Nogués et al., 2015; Caser et al., 2016, 2017). The plant-environment interaction largely contributes to the phytochemicals expression (Kesselmeier and Staudt, 1999; Binns et al., 2002; Dudareva et al., 2013; Holopainen et al., 2013; Selmar and Kleinwächter, 2013; Loreto et al., 2014), and has already been proved to affect the chemical composition of lavender (Prusinowska and Smigielski, 2014). However, the variability in the emission of volatile organic compounds (VOCs) and in the EO biosynthesis is also evidently regulated by genetics, even though the mechanisms involved still need clarifications (Kesselmeier and Staudt, 1999; Sangwan et al., 2001; Brahmkshatriya and Brahmkshatriya, 2013). The intra-specific variation in secondary metabolite production in plants has been reviewed and explained by genetic drift, relaxed selective pressure, introgression of traits through hybridization, gene pleiotropic effects, and phenotypic plasticity (Eckert et al., 2008; Raguso, 2008; Dicke and Loreto, 2010). For instance, qualitatively and quantitatively differences in secondary metabolites have been reported not only between wild and cultivated plants (*L. luisieri*, González-Coloma et al., 2011), but also among plants of the same wild population, as described in lavandin of northwest Italy (Maffei and Peracino, 1993; Peracino et al., 1994) and in *L. latifolia* (Muñoz-Bertomeu et al., 2007).

The populations of plants that are geographically situated at the peripheral limit of a species distribution range are usually expected to exhibit lower abundance and intra-genetic diversity, according to the widely accepted “abundant center model.” Moreover, a higher genetic differentiation than populations at the center is likely to occur (Eckert et al., 2008). Altitudinal gradient was also proved to affect species diversification in peripheral populations of *L. latifolia* (Herrera and Bazaga, 2008). Unfortunately, intra-specific studies on secondary metabolites production performed in plants in different environmental conditions do not inform whether the observed variations represent the genetic adaptation to specific environments, or the short-term response to environmental factors (Spitaler et al., 2006). Thus, trials under uniform cultivation practices are preferable to limit environmental influence on plant secondary metabolite production.

This study aimed to investigate if the environment differences, such as latitude and altitude, affected peripheral population diversification of *L. angustifolia*. Morphology, VOCs, and EOs were analyzed in lavender plants collected along both latitudinal and altitudinal gradients in West Alps (Italy), and grown in pots under uniform conditions, looking for traits of productive interest.

## Materials and methods

### Native environment

**Figure 1:**
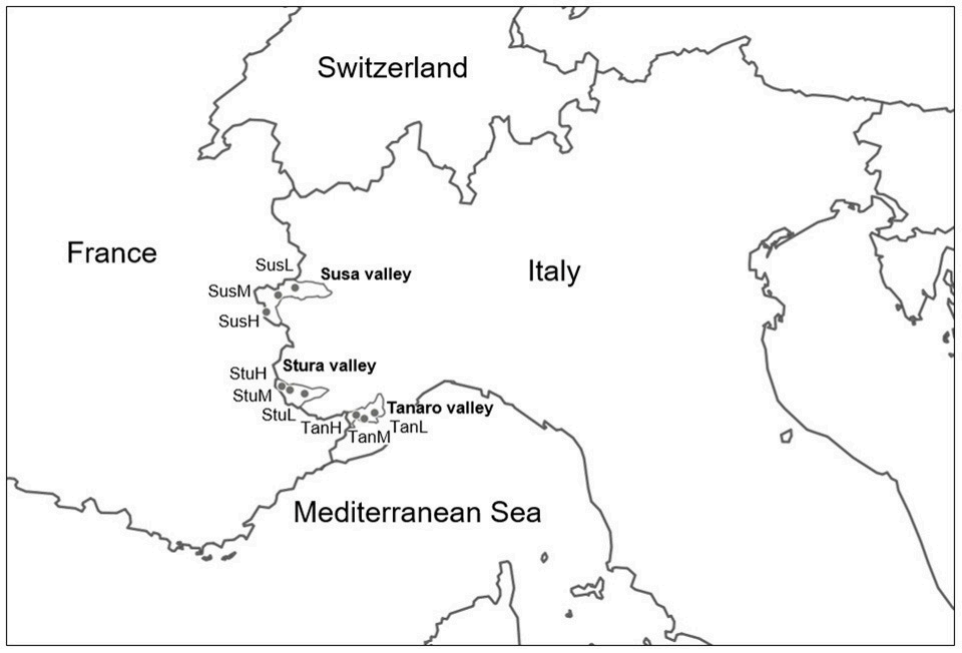
Geographical origin of the nine *L. angustifolia* populations analyzed in this study. For the significance of the population codes, see Table 1.

Lavender (*L. angustifolia*) is widespread throughout Italy (Pignatti, 1982), where it is a common component of low-growing shrub vegetation on calcareous soils. *Lavandula angustifolia* finds its phytosociological optimum in the sub-Mediterranean region within the order *Ononidetalia*, that describes meso-xerophile, basophile, supra-oromediterranean vegetation communities (Theurillat et al., 1995). It is the only species naturally occurring in the West Italian Alps (Piedmont region). Nine peripheral populations (Figure 1) were selected according to the natural range of distribution of the species (Pignatti, 1982), in order to explore different ecological conditions (Table 1, Supplementary Figure 1) in terms of latitude (Susa Valley in the northern range, Stura Valley in the core areas, and Tanaro Valley in the southern part of the Piedmont region) and altitude (low, medium and high altitude). Susa Valley represents the highest latitudinal range of distribution of the species in the Alps (Aeschimann et al., 2004). As the latitude decreases (from Susa to Tanaro Valley), the annual rainfalls decrease and yearly temperatures increase, thus there is a clear ecological gradient moving toward the typical Mediterranean conditions (Fick and Hijmans, 2017).

**Table 1:** Geographical characteristics and climatic conditions of *L. angustifolia* sampling sites in Piedmont region (West Italian Alps).

**Code**	**Valley**	**Latitude (WGS84/32N)**	**Longitude (WGS84/32N)**	**Altitude (m a.s.l.)**	**Mean yearly temperature (°C)**	**Annual rainfall (mm year^−1^)**	**Precipitation of warmest quarter (mm June, July, August)**
SusL	Susa	5001086	349052	910	9.8	859	202
SusM		4994234	334172	1260	7.7	976	213
SusH		4979650	323991	1570	4.8	1,208	232
StuL	Stura	4908484	357570	890	9.8	857	153
StuM		4914856	337635	1360	6.3	1,015	175
StuH		4911955	344807	1810	3.8	1,107	185
TanL	Tanaro	4891985	418508	600	10.4	867	136
TanM		4882887	406442	1240	7.8	969	154
TanH		4890065	402171	1710	5.8	1,036	170

### Plant material and pot cultivation

Portions of lavender branches were collected from wild plants during the flowering season of 2014, from August to September. In each of the nine selected sites, the main area of lavender distribution was identified as a central point and from there, an area with a radius of 10 m was plotted. Inside that area, 10 individuals were randomly chosen and twenty cuttings were immediately prepared from each individual. Specimens are currently available and cultivated in the DISAFA facilities (45°03′58.5″N; 7°35′29.1″E, WGS84 System). Cuttings were allowed to root in peat substrate under plastic tunnels in an organic nursery, specialized in medicinal and aromatic plants (Frat.lli Gramaglia, Collegno, Italy; 45°05′22.4″N, 7°34′26.4″E, 302 m. a.s.l.). In spring 2015, the percentage of successful rooting was calculated and the rooted plants were transplanted in pots, with a mixture of peat and green compost (70-30, % v/v) and transferred outdoors. Irrigation was provided when needed and manual weed control was performed. Plants were fertigated with NPK fertilizer (1:2:2) three times during spring and once during autumn. The cultivation cycle lasted two years and during the first one (Summer 2015), non-destructive VOC analysis was performed *in vivo* on flowered pot plants to characterize lavender aroma and evaluate their potential differences. As ecological characteristics of the sampling site did have an influence on lavender performance, during the second year (Summer 2016), biometric parameters and EOs were analyzed.

### Plant performance evaluation

Biometric and morphological characteristics of 123 rooted flowered plants were evaluated once a week from the beginning of blooming, according to and implementing the guidelines of the International Union for the Protection of New Varieties of Plants (UPOV) proposed for lavenders. The selected parameters were: number of spikes per plant (n), spike length (cm), flowering time (% of flowered plants in each population per week), together with plant height (cm) and width (cm), used to calculate the Growth Index (i.e., the plant volume; cm^3^) according to Demasi et al. (2017). The survival percentage was evaluated at the end of the second cultivation cycle.

### VOCs detection and analysis

Emitted volatiles were analyzed using a Supelco (Bellofonte, USA) SPME device coated with polydimethylsiloxane (PDMS, 100 μm) in order to sample the headspace of each living/flowering plant. Plants of StuM population did not bloom, thus data on VOCs concerned eight populations, for a total of 33 plants. Sample was introduced individually into a 30 ml glass conical flask and allowed to equilibrate for 30 min. After the equilibration time, the fiber was exposed to the headspace for 15 min at room temperature; once sampling was finished, the fiber was withdrawn into the needle and transferred to the injector of the GC system, where the fiber was desorbed. GC-FID and GC-MS analyses were performed according to Pistelli et al. (2017) using a Varian CP-3800 apparatus (Paolo Alto, California, USA) equipped with a DB-5 capillary column (30 m × 0.25 mm i.d., film thickness 0.25 μm) and a Varian Saturn 2000 ion-trap mass detector. The oven temperature was programmed rising from 60°C to 240°C at 3°C/min; injector temperature, 220°C; transfer-line temperature, 240°C; carrier gas, He (1 ml/min).

### Essential oil extraction and analysis

Essential oil of lavender plants was obtained by hydro distillation of dried aerial parts of each sample using a Clevenger-type apparatus according to the Italian Pharmacopoeia (Helrich, 1990). Flowered plants of StuM population did not produce enough material to obtain EO, thus data on EOs concerned eight populations, for a total of 122 plants. The obtained EOs were immediately injected in GC-FID and GC-MS according to the method described in Pistelli et al. (2017). Identification of the constituents was based on the comparison of the retention times with those of authentic samples in comparing their linear retention indices (*LRI*) relative to a series of n-hydrocarbons, and on computer matching against commercial (Adams, 2007; NIST/EPA/NIH Mass Spectral Library, 2014) and home-made mass spectra library built up from pure substances and components of known EOs as well as MS literature data.

### Statistical analyses

Data were tested for the homogeneity of variance, then one-way ANOVA was performed to analyze latitude and altitude effect on biometric parameters and means were separated according to Ryan-Einot-Gabriel-Welsch-F post-hoc test (REGW-F). Statistically significant differences induced by latitude on chemical classes and compounds of VOCs and EO were assessed with the one-way ANOSIM with Euclidean-based similarity. The percentage contribution of each compound to the observed dissimilarity was assessed through the similarity percentage analysis (SIMPER, Euclidean distance). For each compound the difference between valleys were tested with Mann-Whitney pairwise test. Besides, VOC and EO chemical classes were subjected to the regression analysis to investigate their eventual link to altitude or latitude of native environment. The value for statistical significance was *P* < 0.05. The ANOVA analysis was performed with SPSS software (version 22), while all other analyses were performed with Past software (version 3).

## Results and discussion

### Morphology and performance of cultivated lavender

**Figure 2:**
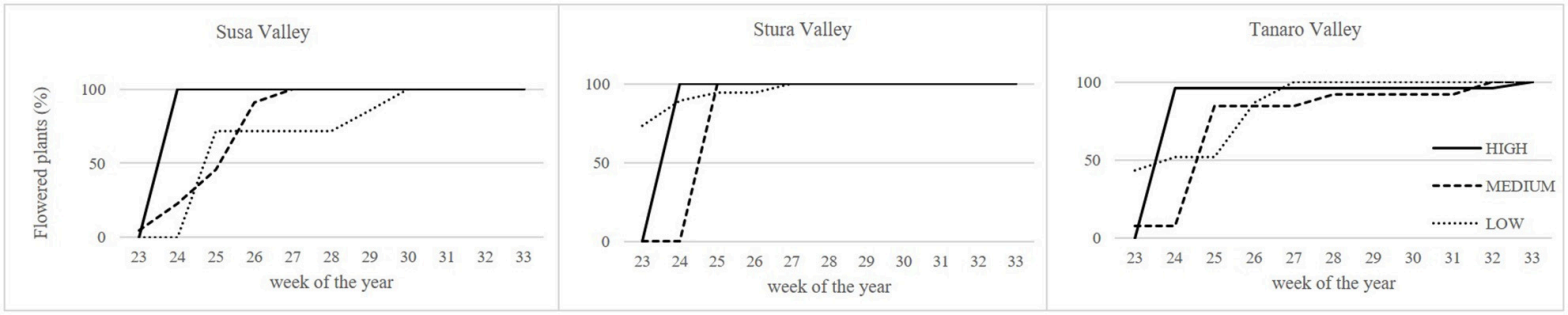
Blooming trend of lavender plants during the second cultivation cycle under uniform conditions. Solid lines, higher altitudes; dashed lines, medium altitudes; dotted lines, lower altitudes.

Lavenders of West Alps differently performed under cultivation, and influence of sea distance (latitude) or altitude of the native environment was observed in some biometric parameters (Table 2). Rooting percentage after 3 months in seed cells was generally below 40% and ranged among populations (SusH 23.9%, SusM 17.0%, SusL 38.0%; StuH 15.8%, StuM 9.6%, StuL 35.3%; TanH 37.5%, TanM 23.0%, TanL 39.5%). Rooting ability was therefore generally low, especially considering that selected lavender cultivars often reach 95% of successful rooting (Lis-Balchin, 2003). However, the propagation of wild adult plants by cutting is needed to produce genetically homogeneous individuals and the rooting percentage achieved in this study on *L. angustifolia* is considered of interest by local nurseries. Significant effects were observed due to both altitude and latitude of native environment, with a more successful rooting in plants evolved closer to the Mediterranean conditions (Tanaro) and from lower altitudes. The survival rate of rooted cuttings after 2 years of cultivation ranged between 54% (SusL) and 83% (StuL), but was independent from the geographical origin of the plants (Table 2). During the first cultivation cycle, 9% of pot lavenders bloomed, while in the second year, all plants bloomed (Figure 2); the first flowered plants were recorded on June 6th 2016 and the last on August 19th 2016. Interestingly, almost all lavenders of high populations flowered simultaneously in 1 week (SusH 100%, StuH 100%, and TanH 96%) regardless the latitude, followed by medium and low altitude populations, with the latter group defining a more gradual trend during summer. Knowledge of flowering times is essential to plan nursery activities (Lis-Balchin, 2003) or harvesting time for the production of EOs or dried flowers. Indeed, the nurseries can exploit lavenders with a flower production concentrated in a short period, since higher number of plants are available per sale. Furthermore, producers can optimize flower harvesting in the field when a simultaneous blooming occurs. Nonetheless, a gradual blooming can ensure flowered plants availability in the nursery and gardens for a longer period.

**Table 2:** Differences in rooting percentage, survival rate, growth index (GI), spike number and spike length recorded in the cultivated lavenders, according to altitude (High, Medium, and Low) and latitude (Susa, Stura, and Tanaro) of the native environment.

	**Rooting (%)**	**Survival (%)**	**GI (cm^3^)**	**Spike (n)**	**Spike length (cm)**	**Flower yield (g DW plant^−1^)**
**ALTITUDE**						
High	25.7 b^§^	67	2003 b	11.26 a	37.8 a	1.16 b
Medium	16.5 c	64	2125 ab	6.19 b	28.9 b	1.84 a
Low	37.6 a	69	3005 a	8.53 b	24.9 b	1.92 a
*P*	^***^	ns	^*^	^**^	^***^	^*^
**LATITUDE**						
Susa	26.3 b	66	1886 b	5.4 b	27.5	1.38
Stura	20.2 b	73	3706 a	10.6 a	30.5	1.84
Tanaro	33.3 a	61	2470 b	8.8 a	30.4	1.79
*P*	^*^	ns	^**^	^**^	ns	ns

§ *Means followed by the same letter in the same column denote no significant differences according to REGW-F test (P < 0.05). ns*,

*,** or *** *indicates non-significant differences or significant at P ≤ 0.05, 0.01, or 0.001, respectively*.

Altitude affected also morphological parameters and flower yield (Table 2). Contrariwise to low altitude groups, plants from higher elevation developed a more compact shrub (GI = 2,003 cm^3^), with higher number (11.26) and longer spikes (37.8 cm) during cultivation, but producing lower yield of inflorescences (1.16 gDW/plant), attributable to a less number of flowers per spike. Altitudinal gradient effect has been previously observed in other Lamiaceae species, displaying lower height at high altitudes, to avoid wind damages and improve photosynthetic conditions (Kofidis and Bosabalidis, 2008 and references in it). However, compact plants can facilitate field operations, especially harvesting. Growth and number of flower spikes were influenced also by the population distance from the sea. Plants from Susa Valley showed a compact growth (GI = 1,886 cm^3^) and a low number of flower spikes (5.4), contrary to what observed in Stura plants (GI = 3,706 cm^3^ and 10.6). Tanaro plants were compact (GI = 2,470 cm^3^) and produced a high number of spikes (8.8), showing an interesting habit for ornamental and field cultivation purposes. A latitudinal gradient in spike length and flower yield were not detected in the second cultivation cycle. These data could be helpful in the selection of alpine peripheral *L. angustifolia* populations with interesting traits for nurseries both for ornamental and production purposes.

### Influence of native environment on volatile emission

The aroma profile represents the volatiles spontaneous emitted from the living plants and is a highly complex component of floral phenotype (Raguso, 2008). High intra and inter-specific variations in secondary metabolite production and aroma composition were reported in *Lavandula* genus, including *L. latifolia* and *L. luisieri* (Sanz et al., 2004; Muñoz-Bertomeu et al., 2007; González-Coloma et al., 2011) and other ornamental plants, such as rose, magnolia, and butterfly bush (Azuma et al., 2001; Raguso, 2008; Gong et al., 2014).

**Figure 3:**
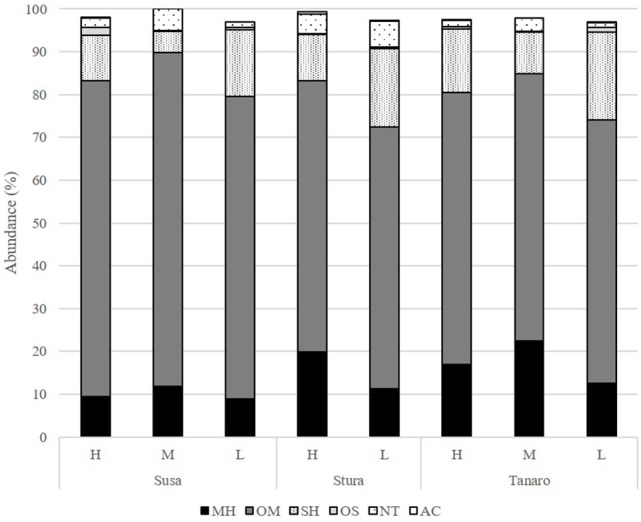
Chemical classes composition (%) of the *L. angustifolia* aroma after one cultivation cycle in uniform conditions. H, high altitude; M, medium altitude; L, low altitude; MH, monoterpene hydrocarbons; OM, oxygenated monoterpenes; SH, sesquiterpene hydrocarbons; OS, oxygenated sesquiterpenes; NT, non terpene derivatives; AC, apocarotenoids.

**Figure 4:**
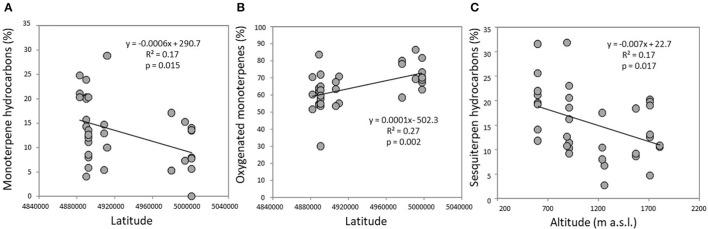
Significant linear regressions detected in the headspace of flowering lavender plants between **(A)** monoterpene hydrocarbons and latitude; **(B)** oxygenated monoterpenes and altitude; **(C)** sesquiterpene hydrocarbons and altitude.

The volatile chemical classes of lavender fragrance were mainly represented by terpenes, with low amount of non-terpene derivatives (NT, 1.1–6.1%) and apocarotenoids (AC, 0.1–0.5%) (Figure 3, Supplementary Table 1). Oxygenated monoterpenes were the most abundant VOCs in all the populations (OM, 61.2–78.1%), followed by monoterpene hydrocarbons (MH, 8.9–22.4%), sesquiterpene hydrocarbons (SH, 4.8–20.5%), and oxygenated sesquiterpenes (OS, 0.1–1.9%). Interestingly, SH were generally present in higher amounts in the aroma of the lowest-altitude populations (SusL 15.6%, StuL 18.3%, TanL 20.5%), and AC were not detected in SusM, SusL, and in TanM populations. One-way ANOSIM performed on chemical classes showed that populations far from the sea (Susa) significantly differed from the closest ones (Tanaro) in their phytochemical composition (*p* = 0.038), indicating a clear distinction of aroma profiles. Moreover, a linear negative regression fitted between latitude and MH and a positive with OM (Figures 4A,B), whereas a negative regression model fitted between SH and altitude (Figure 4C). Monoterpenes are high volatile terpenoids emitted with warm temperature (>20°C). Their amount in flower fragrance is variable and often exceeds 50% of the total flower emission (Holopainen et al., 2013), as found in our study. Sesquiterpenes are the most diverse class of terpenoids (Holopainen et al., 2013); they are more abundant in plant volatiles than oils and are effectively used in aromatherapy (Cavanagh and Wilkinson, 2002), giving a sweet aromatic note (Prusinowska and Smigielski, 2014). Therefore, altitude influence on sesquiterpenes content found in this study could be an interesting starting point to explore the selection of fragrance characteristics.

Considering single compounds, 88 VOCs were identified in lavender aroma profile (Supplementary Table 1). The identified compounds accounted for more than 97% of the total composition. The number of compounds in each population varied from 42 (TanM) to 62 (StuL). Interestingly, every population had a peculiar aroma composition and all populations shared only 27 compounds. Among these compounds, the sum of linalyl acetate, linalool and 1,8-cineole were present more than 50% in SusM and SusL populations and more than 40% in the others; 1,8-cineole was less abundant in TanM and TanL; 4-terpineol and (E)-β-ocimene occurred in high percentages only in TanM lavender (7.7% and 7.5%, respectively), which showed also higher amount of lavandulyl acetate (4.5%). Linalool (22.9%), β-caryophyllene (13.9%), as well as borneol (6.7%) evidenced the highest amount in TanL. Da Porto and Decorti (2008) found a different arrangement in VOCs of lavender cultivated in Northeast Italy, in particular with 1,8-cineole, linalool, camphor, linalyl acetate, and β-caryophyllene being the 70% of the total aroma. Moreover, (E)-β-ocimene was present in smaller amounts (0.2–0.7%) compared to our study (1.6–7.5%).

The one-way ANOSIM performed using VOCs composition showed a similar trend to what observed in chemical classes, with a clear differentiation between Susa and Tanaro populations (*p* = 0.018), respectively the farthest and the closest sites to Mediterranean conditions. According to the SIMPER analysis, 11 compounds were responsible for the 95% of this dissimilarity in lavender fragrance (Table 3), with 1,8-cineole, linalyl acetate and linalool as major contributors (50.65, 16.33, and 13.16% of the contribution, respectively). The effect of sea distance was particularly seen on five out of 11 compounds. In detail, linalool, (E)-β-ocimene and 4-terpineol increased in the populations closer to the Mediterranean Sea, whereas 1,8-cineole and bornyl acetate had an opposite behavior. These latter constituents usually adversely affect lavender fragrance (Prusinowska and Smigielski, 2014), while linalool is responsible for the fresh and floral smell (Chizzola, 2013; Prusinowska and Smigielski, 2014). It is one of the best-examined and most often reported monoterpene in flowers (Buchbauer and Ilic, 2013; Holopainen et al., 2013). Moreover, there are evidences that linalool acts synergistically with linalyl-acetate and both compounds are required for the anxiolytic effect of the inhaled EOs (Buchbauer and Ilic, 2013). Thereof peripheral populations adapted to Mediterranean ecological conditions (Tanaro) appear more interesting for the selection of fragrance genotypes.

**Table 3:** List of VOCs responsible for dissimilarity induced by latitude in lavender aroma according to the SIMPER analysis.

**Compound**	**Contribution (%)**	**Cumulative (%)**	**Susa**	**Stura**	**Tanaro**	**Sign**
1,8-cineole	50.7	50.7	22.26a^§^	15.38ab	9.84b	^*^
Linalyl acetate	16.3	67.0	21.12	17.14	16.30	ns
Linalool	13.2	80.2	9.85b	11.62ab	16.35a	^*^
β-caryophyllene	4.4	84.6	9.16	8.90	10.98	ns
Borneol	2.5	87.1	4.86	3.66	5.83	ns
(E)-β-ocimene	1.8	88.9	2.18b	4.28ab	4.72a	^*^
(E)-β-farnesene	1.5	90.4	0.80	3.04	1.61	ns
(Z)-β-ocimene	1.5	91.9	2.34	4.84	4.08	ns
4-terpineol	1.5	93.4	1.11b	3.38ab	3.55a	^*^
Bornyl acetate	1.1	94.5	3.36*a*	0.80ab	0.36b	^**^
Lavandulyl acetate	0.8	95.3	1.72	2.48	2.50	ns

§ *Data followed by the same letter in the same line are not significantly different according to Mann-Whitney test. ns*,

*, ** or *** *indicates non-significant differences or significant at P ≤ 0.05, 0.01 or 0.001, respectively*.

Both isomers of β-ocimene, together with linalool, are frequently found in species pollinated by butterflies, bees, and moths (Andersson, 2003; Dötterl and Schäffler, 2007; Gong et al., 2014). TanM population emitted the highest quantity of both isomers [(Z)-β-ocimene, 6.4% and (E)-β-ocimene, 7.5%] and could have therefore differentiated its VOC emission to attract its peculiar type of pollinators (Dudareva et al., 2013).

Qualitative and quantitative variation was found in this study in the fragrance composition of *L. angustifolia* species according to its distance from the sea, while Stark et al. (2008) found only qualitative differences concerning other secondary metabolites (flavonoids and tannins). Several phenological events and environmental parameters may drive intra-specific floral scent variation, as observed also between and within *Phlox* cultivars (Majetic et al., 2014), highlighting that species contain a great potential of variation. Nonetheless, the uniform cultivation conditions adopted in our trial pointed out that variation in lavender fragrance composition could be partly attributed to its genetic adaptation to ecological conditions of peripheral alpine sites.

### Influence of native environment on EO composition

Generally, EO yields were consistent with previous studies on *L. angustifolia* (Da Porto et al., 2009; Prusinowska and Smigielski, 2014) which are very low in SusH and TanM to reach 0.69% in SusL (Supplementary Table 2). Plants from the lowest altitudes showed higher yields in Susa and Tanaro valley (0.69 and 0.37%, respectively). Previous studies highlighted the same behavior in other aromatic plants such as *Origanum vulgare* (Vokou et al., 1993; Giuliani et al., 2013), *Coriandrum sativum* (Shams et al., 2016), and *Rosmarinus officinalis* (Tuttolomondo et al., 2015). Giuliani et al. (2013) found a correlation between higher yields at decreasing altitudes and, but not limited to, an increased presence of glandular peltate trichomes, the structures responsible for the accumulation of EOs. Considering distance from Mediterranean conditions, Susa lavenders were the most productive in terms of EO, followed by Tanaro and Stura plants.

**Figure 5:**
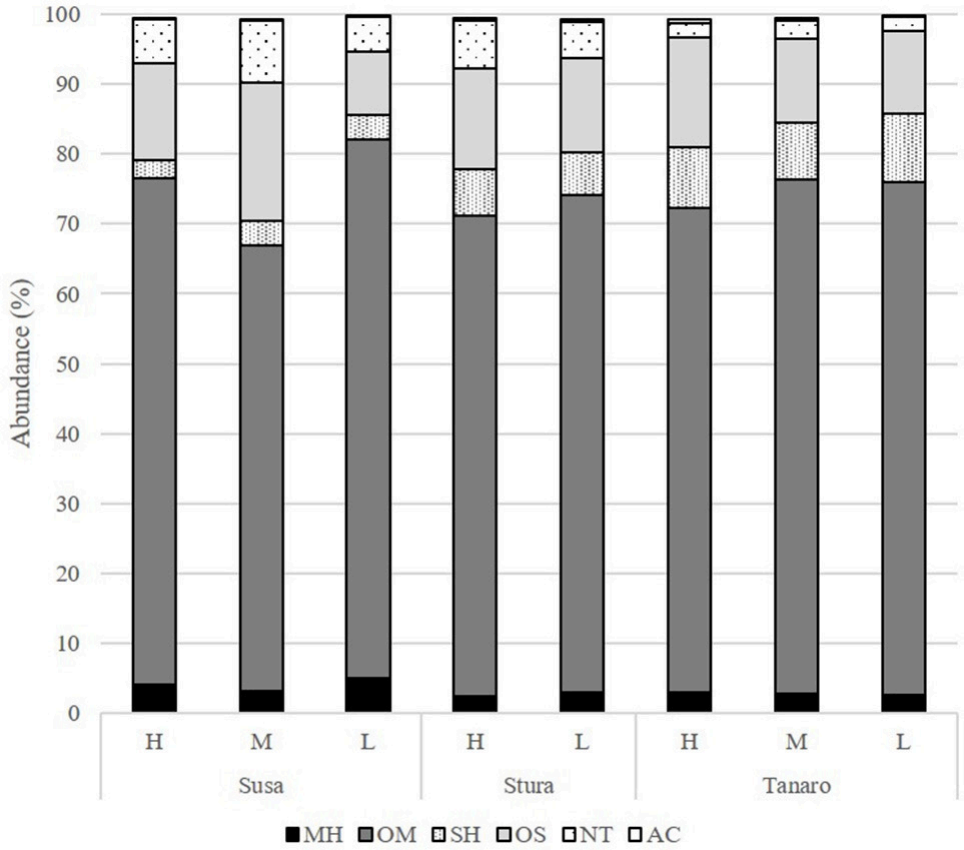
Chemical classes composition (%) of the *L. angustifolia* essential oils after two cultivation cycle in uniform conditions. H, high altitude; M, medium altitude; L, low altitude; MH, monoterpene hydrocarbons; OM, oxygenated monoterpenes; SH, sesquiterpene hydrocarbons; OS, oxygenated sesquiterpenes; NT, non terpene derivatives; AC, apocarotenoids.

**Figure 6:**
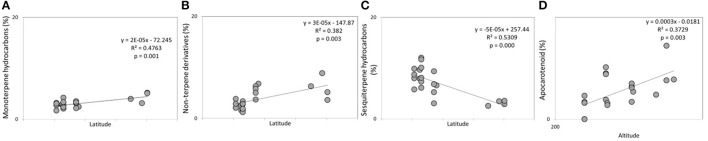
Significant linear regressions detected in lavender EOs between **(A)** monoterpene hydrocarbons and latitude; **(B)** non-terpene derivatives and latitude; **(C)** sesquiterpene hydrocarbons and latitude; **(D)** apocarotenoids and altitude.

The mean of the relative abundance of chemical classes was distributed as follows (Figure 5, Supplementary Table 2): OM (71.2%), OS (13.7%), SH (6.1%), NT (4.9%), MH (3.2%), and AC (0.3%). A different distribution was recorded by Pistelli et al. (2017) in *L. angustifolia* ‘Maillette’, where the amount of OS and SH were lower than that observed in this study (0.5 and 1.8%, respectively). Commonly, monoterpenes represent the major class in the oil of *Lavandula* species (Lis-Balchin, 2003) and our data are consistent with values reviewed by Prusinowska and Smigielski (2014). In this study, significant positive linear regression fitted between MH and NT concentration with latitude (Figures 6A,B) and AC content with altitude (Figure 6D); instead, a negative linear regression fitted between SH and latitude (Figure 6C), an opposite behavior than that observed in *Teucrium polium* EO by Sadeghi et al. (2014).

One-hundred-four compounds were identified in lavender EO (Supplementary Table 2), accounting for more than 99% of the total EO composition. The number of compounds in each population varied from 63 (SusH) to 83 (StuL). These numbers were higher than those previously reported in *L. angustifolia* species and cultivars cultivated in the Tyrrhenian coast (Venskutonis et al., 1997; Pistelli et al., 2017). Interestingly, each EO was a mixture of different compounds, but all populations shared at least 40 constituents. The EO composition of *L. angustifolia* is reported to be the most variable inside the genus (Lis-Balchin, 2003). However, the characteristic compounds found in *Lavandula* oil usually are linalool, linalyl acetate, 1,8-cineole, β-ocimene, 4-terpineol, and camphor (Cavanagh and Wilkinson, 2002). Accordingly, in the present study linalool and linalyl acetate were the most abundant in all the EOs analyzed, with the highest amount recorded in TanL (linalool 35.0%) and StuH (linalyl acetate 26.3%). However, linalool content was present in lower percentage than in previous studies on lavender (Venskutonis et al., 1997; Pistelli et al., 2013, 2017). In fact, linalool production in *L. angustifolia* varies widely (Prusinowska and Smigielski, 2014) depending on temperature, flower development, and rainfall (Hassiotis et al., 2014). Linalyl acetate content registered in this work is in accordance with that reported for lavender ‘Maillette’ (Pistelli et al., 2017), but lower than in lavender species grow in Lithuania (Venskutonis et al., 1997). According to the percentage content of the characteristic compounds of lavender essential oil reported in the European Pharmacopoeia 6.0 (Ph. Eur.) none of the analyzed EOs completely fulfill the expected limits. StuH and TanL were closely related to the established ranges except for linalool (less than 20%) in StuH and linalyl acetate (less than 25%) in TanL. The percentages of 4-terpineol, 3-octanone, limonene, and lavandulyl acetate were in the range fixed by Ph. Eur. for all the tested EOs. Among the compounds reported in ISO 3515:2002 for lavender EO^1^ only 3-octanone, limonene and lavandulyl acetate respected the fixed limits in all the analyzed EOs. The analysis of flower fragrance has been suggested as a good indicator of the EO composition (An et al., 2001). However, EO composition frequently differed from the aroma profiles (see section Influence of Native Environment on Volatile Emission), as already highlighted in several studies on lavenders (Cavanagh and Wilkinson, 2002). These differences were caused by processes such as hydrolysis, thermal degradation, and molecular rearrangements that are promoted during the hydro distillation method (Da Porto and Decorti, 2008).

The one-way ANOSIM performed on EO compounds revealed significant differences among populations at different latitudes (*p* = 0.009), likewise to what observed in lavender aroma (see section Influence of Native Environment on Volatile Emission), with a clear separation between populations with more (Tanaro) and less (Susa) Mediterranean ecological conditions. According to the SIMPER analysis, thirteen compounds were responsible for 95% of this difference (Table 4), with linalool being the major contributor (45.98%). The effect of sea distance was seen on nine out of the 13 compounds. In particular, linalool, 4-terpinenol, β-caryophyllene, thujapsan-2-α-ol, and (Z)-γ-bisabolene were higher in populations closer to the Mediterranean Sea (Tanaro), while 1,8-cineole, τ-cadinol, 1-octen-3-yl acetate and α-terpineol where higher in the furthest populations (Susa). Linalool and terpineol are of great interest for medicinal industry due to their positive effects on the central nervous system (Prusinowska and Smigielski, 2014). β-caryophyllene, with its woody and spicy aroma, is commonly used in the fragrance and cosmetic industry. However, it has also antibiotic, anesthetic, anti-inflammatory, antioxidant, and anti-spasmodic activity (Buchbauer and Ilic, 2013). Therefore, similarly to results on fragrance, the populations closer to Mediterranean conditions (Tanaro) could be considered valuable germplasm for selection and breeding purposes, aimed to the production of EO, despite the low yields obtained. Indeed it is needed to consider that the cultivation occurred in pot. In proper open field conditions higher biomass and EO yields are expected. No significant differences were detected in EO compounds according to altitude, likewise to VOCs composition (see Section Influence of Native Environment on Volatile Emission), despite there are evidence that altitude significantly affected chemical composition of EO in other Lamiaceae plants (Giuliani et al., 2013; Sadeghi et al., 2015). Chograni et al. (2010) observed also a phytochemical variation in the leaf EO of wild *L. multifida* belonging to the same bioclimatic zone, possibly explained by genetic factors. Similarly, in the present study, the ecological conditions of peripheral sites may have induced different adaptation mechanisms in lavender, leading to different phytochemical compositions. Since the EO obtained in this work did not respect the limit established by Ph. Eur., they can be used for other industrial application, or for cosmetic purposes.

**Table 4:** List of compounds responsible for dissimilarity induced in lavender EO by latitude according to the SIMPER analysis.

**Compound**	**Contribution %**	**Cumulative %**	**Susa**	**Stura**	**Tanaro**	**Sign**.
Linalool	46.0	46.0	20.97b^§^	23.95ab	29.50a	^*^
Linalyl acetate	17.7	63.7	17.67	15.27	14.88	ns
1,8-cineole	9.2	72.8	6.29a	1.50b	1.01b	^***^
Lavandulyl acetate	3.7	76.5	3.29	3.93	3.82	ns
Caryophyllene oxide	3.5	80.0	6.09	7.39	6.58	ns
T-cadinol (= epi-α-cadinol)	3.5	83.5	3.03a	0.65b	0.16b	^**^
Borneol	3.2	86.7	4.38	5.28	4.61	ns
4-terpinenol	2.3	89.0	0.87b	1.43ab	3.01a	^*^
1-octen-3-yl acetate	2.2	91.2	3.01a	2.38a	0.60b	^*^
β-caryophyllene	1.4	92.6	1.31b	1.83b	3.16a	^**^
Thujapsan-2-α-ol	0.8	93.4	0.03b	1.40a	1.49a	^*^
(Z)-γ-bisabolene	0.7	94.2	0.53b	1.89a	1.89a	^*^
α-terpineol	0.6	94.8	4.10a	3.05ab	2.90b	^*^

§ *Data followed by the same letter in the same line are not significantly different according to Mann-Whitney test. ns*,

*,** or*** *indicates non-significant differences or significant at P ≤ 0.05, 0.01 or 0.001, respectively*.

## Conclusions

We hereby provide the first phytochemical description of secondary metabolites in *L. angustifolia* peripheral populations of the West Italian Alps. The influence of sea distance (latitude) and altitude on VOC and EO composition was analyzed in plants grown under uniform cultivation conditions. Interestingly, results showed that the ecological gradient created germplasm heterogeneity. Compared to other studies, altitude seemed to affect mainly biometric parameters, and to a low extent the phytochemical composition of *L. angustifolia*, while sea distance had an influence on both morphological and phytochemical traits. Lavenders of West Italian Alps disclosed a great potential for the development of a valuable local product, where the cultivation of the thermophilous species *L*. × *intermedia* cannot be applied due to the lower average temperature of the studied alpine areas. Among the studied alpine peripheral populations, plants of Tanaro Valley, which have evolved in almost Mediterranean ecological conditions, generally performed better in both morphological and phytochemical characteristics. To obtain uniform productions, the study of the phytochemical variation and the selection and propagation of interesting genotypes must be promoted, together with the improvement of cultivation protocols.

## Author contributions

SD and MC equally contributed to this work in the acquisition of data, analysis and interpretation of data, and drafting of manuscript. BN and PC carried out phytochemical analyses. ML contributed to lavender population selection and data analysis. LP and BN contributed to data interpretation and discussion. VS conceived, designed and coordinated the work. LP and VS critically revised and approved the manuscript.

### Conflict of interest statement

The authors declare that the research was conducted in the absence of any commercial or financial relationships that could be construed as a potential conflict of interest.

## Footnotes

^1^ISO 3515:2002 Oil of Lavender (*Lavandula angustifolia* Mill.).

## Abbreviations

AC: Apocarotenoids
EO: Essential Oils
GC-MS: Gas Cromatography-Mass Spectrometry
GI: Growth Index
MH: Monoterpene Hydrocarbons
NT: Non Terpene Derivatives
OM: Oxygenated Monoterpenes
OS: Oxygenated Sesquiterpenes
SH: Sesquiterpene Hydrocarbons
SPME: Solid Phase Micro Extraction
VOCs: Volatile Organic Compounds.
