# Supplementary material for: Latitude and Altitude Influence Secondary Metabolite Production in Peripheral Alpine Populations of the Mediterranean Species Lavandula angustifolia Mill
Source: Front Plant Sci. 2018 Jul 5;9:983. doi: 10.3389/fpls.2018.00983 (PMC6042283; doi:10.3389/fpls.2018.00983)
Supplement: Supplementary file 1 [file Data_Sheet_1.docx]

***Supplementary Material***

**Latitude and altitude influence secondary metabolite production in peripheral alpine populations of the Mediterranean species *Lavandula angustifolia* Mill.**

**Sonia Demasi, Matteo Caser, Michele Lonati, Pier Luigi Cioni, Luisa Pistelli, Basma Najar, Valentina Scariot^*^**

^*^**Correspondence**: Valentina Scariot, valentina.scariot@unito.it

# Supplementary Figures


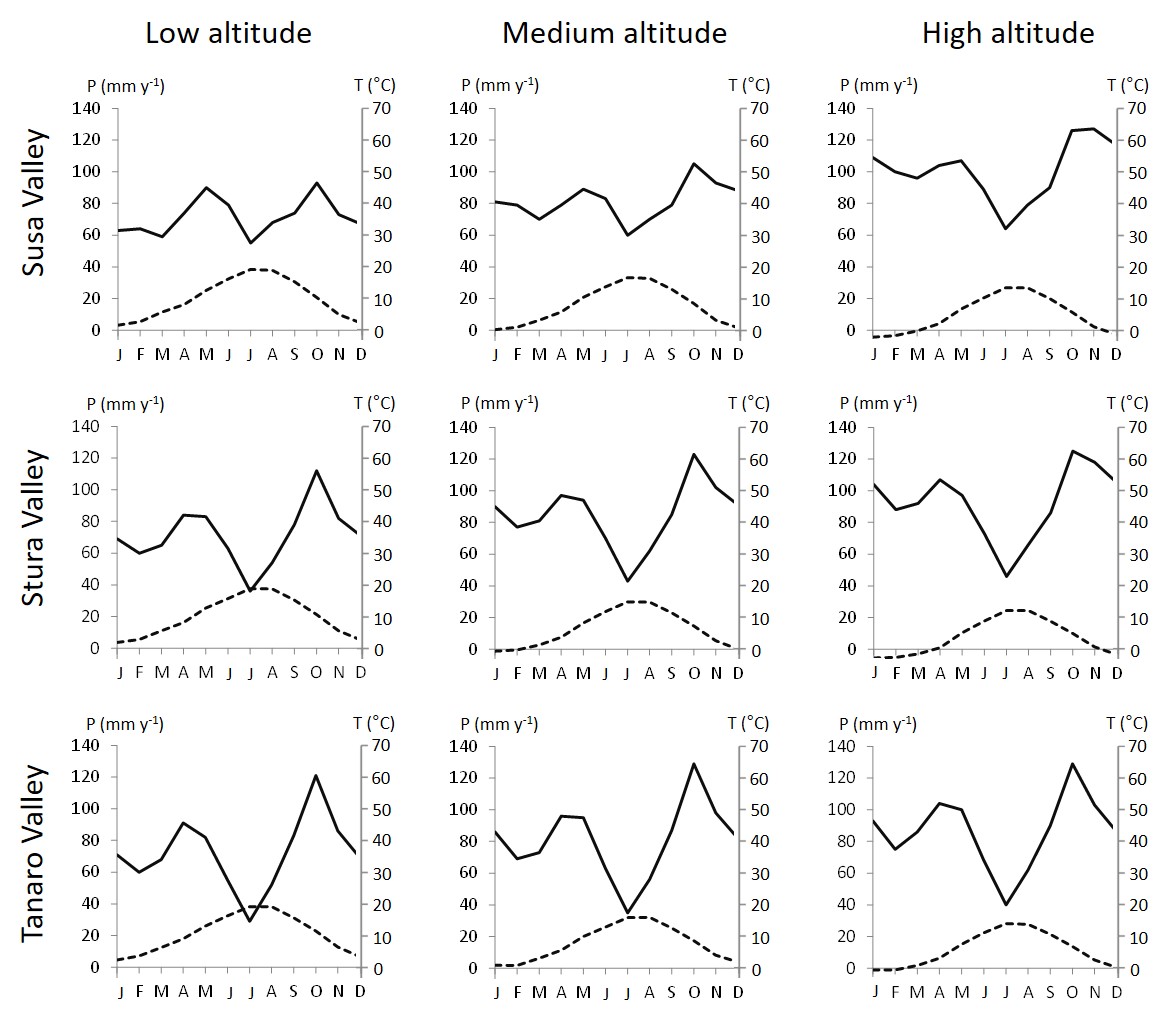


**Supplementary Figure 1.** Climatic diagram of the nine sites identified for *L. angustifolia* sampling in West Italian Alps, according to Bagnouls and Gaussen (1957).

Bagnouls, F., and Gaussen, H. (1957). Les climats biologiques et leur classification. *Ann. Georgr.*, 193–220. doi:10.2307/23443505.
