# Supplementary material for: Latitude and Altitude Influence Secondary Metabolite Production in Peripheral Alpine Populations of the Mediterranean Species Lavandula angustifolia Mill
Source: Front Plant Sci. 2018 Jul 5;9:983. doi: 10.3389/fpls.2018.00983 (PMC6042283; doi:10.3389/fpls.2018.00983)
Supplement: Supplementary file 2 [file Data_Sheet_2.docx]

***Supplementary Material***

**Latitude and altitude influence secondary metabolite production in peripheral alpine populations of the Mediterranean species *Lavandula angustifolia* Mill.**

**Sonia Demasi, Matteo Caser, Michele Lonati, Pier Luigi Cioni, Luisa Pistelli, Basma Najar, Valentina Scariot^*^**

^*^**Correspondence**: Valentina Scariot, valentina.scariot@unito.it

**Supplementary Table 1.** Average abundance (%) of VOCs in the headspace of the eight studied *L. angustifolia* populations, during the first cultivation cycle under uniform growing conditions. The compounds are listed in their elution order on the DB-5 column. Compounds with relative percentages smaller or equal to 0.1% were excluded from the table and the analysis. *LRI_exp_*= experimental linear retention index relative to a series of alkanes; *LRI_lit_*= linear retention index from the literature on DB-5column (Adams, 2007); *LRI*=linear retention index on HP-Wax column. H=high altitude, M=medium altitude, L=low altitude, OM=oxygenated monoterpenes, MH=monoterpene hydrocarbons, SH=sesquiterpene hydrocarbons, OS=oxygenated sesquiterpenes, NT=non terpene derivatives, AC=apocarotenoid.

| Compound | Class | *LRI_exp_* | *LRI_lit_* | *LRI* | Susa | | | Stura | | Tanaro | | |
| --- | --- | --- | --- | --- | --- | --- | --- | --- | --- | --- | --- | --- |
|  |  |  |  |  | **H** | **M** | **L** | **H** | **L** | **H** | **M** | **L** |
| 1-hexanol | NT | 870 | 871 | 1358 |  |  |  | 0.2 | 0.1 |  |  |  |
| tricyclene | MH | 930 | 927 | 1003 |  | 0.1 |  | 1.6 |  |  | 0.4 | 0.1 |
| α-pinene | MH | 939 | 939 | 1015 | 0.2 | 0.2 | 0.1 | 1.1 | 0.2 | 0.4 | 0.6 | 0.3 |
| β-citronellene | MH | 950 | 950 | - |  |  |  | 0.5 |  |  | 0.1 |  |
| camphene | MH | 955 | 954 | 1058 | 1.0 | 1.9 | 1.3 | 1.0 | 0.2 | 1.9 | 0.7 | 2.2 |
| 1-heptanol | NT | 969 | 967 | 1464 |  |  |  |  | 0.1 |  |  |  |
| sabinene | MH | 976 | 975 | 1117 | 0.2 |  | 0.4 | 0.2 |  | 0.1 | 0.1 |  |
| β-pinene | MH | 981 | 979 | 1081 | 0.4 | 0.4 | 0.2 | 0.2 | 0.2 | 0.5 |  | 0.2 |
| 1-octen-3-ol | NT | 978 | 979 | 1447 | 0.1 |  |  |  | 0.2 |  |  |  |
| 3-octanone | NT | 987 | 984 | 1262 | 0.2 | 0.3 |  | 0.5 |  | 0.2 | 0.7 | 0.1 |
| myrcene | MH | 990 | 991 | 1164 | 2.3 | 1.7 | 2.0 | 2.0 | 0.9 | 0.8 | 4.2 | 1.3 |
| 2,3-dehydro-1,8-cineole | OM | 992 | 991 | 1178 |  |  |  |  | 0.2 |  |  |  |
| octanal | NT | 1002 | 999 | 1289 |  |  |  |  | 0.4 |  |  |  |
| α-phellandrene | MH | 1005 | 1003 | 1157 |  |  |  |  | 0.3 |  |  |  |
| N-hexyl acetate | NT | 1013 | 1009 | 1276 | 0.2 | 0.3 |  | 0.1 |  |  |  |  |
| δ-3-carene | MH | 1011 | 1031 | 1142 |  |  | 0.1 |  | 0.1 | 0.3 | 0.7 | 0.1 |
| α-terpinene | MH | 1018 | 1017 | 1177 |  |  |  |  |  | 0.1 | 0.2 |  |
| o-cymene | MH | 1026 | 1026 | 1287 | 0.2 | 0.3 | 0.1 | 0.1 | 0.2 | 0.3 | 0.1 | 0.1 |
| p-cymene | MH | 1028 | 1025 | 1266 | 0.5 | 1.0 | 0.3 | 0.4 | 1.4 | 1.3 | 0.2 | 0.7 |
| limonene | MH | 1031 | 1029 | 1194 |  |  | 0.1 | 0.4 | 0.4 |  |  | 0.9 |
| 1,8-cineole | OM | 1034 | 1031 | 1209 | 20.9 | 28.3 | 21.1 | 9.5 | 19.3 | 22.4 | 2.0 | 2.4 |
| (Z)-β-ocimene | MH | 1041 | 1037 | 1242 | 2.0 | 2.2 | 2.6 | 6.4 | 3.8 | 5.1 | 6.4 | 2.2 |
| (E)-β-ocimene | MH | 1051 | 1050 | 1253 | 2.5 | 3.7 | 1.6 | 5.6 | 3.4 | 5.7 | 7.5 | 2.7 |
| γ-terpinene | MH | 1062 | 1060 | 1238 |  |  |  | 0.2 |  | 0.1 | 0.5 | 1.4 |
| cis-sabinene hydrate | MH | 1068 | 1070 | 1463 | 0.2 | 0.3 | 0.1 | 0.2 | 0.1 | 0.3 | 0.4 | 0.4 |
| cis-linalool oxide (furanoid) | OM | 1075 | 1087 | 1457 | 1.1 | 0.6 | 1.1 | 0.3 | 0.5 | 0.6 | 0.3 | 0.4 |
| camphenilone | OM | 1083 | 1082 | 1474 |  | 0.2 |  |  |  |  |  |  |
| p-mentha-2,4(8)-diene | MH | 1086 | 1088 | 1637 |  |  |  |  | 0.1 |  |  |  |
| terpinolene | MH | 1088 | 1089 | 1276 |  |  |  |  |  |  | 0.3 |  |
| trans-linalool oxide (furanoid) | OM | 1089 | 1073 | 1461 | 0.8 | 0.2 | 0.9 | 0.2 | 0.4 | 0.4 | 0.2 | 0.6 |
| 6-camphenone | OM | 1094 | 1097 | - | 0.1 |  |  |  | 0.2 | 0.1 |  |  |
| linalool | OM | 1099 | 1097 | 1550 | 5.8 | 7.3 | 12.3 | 14.2 | 9.9 | 9.2 | 15.4 | 22.9 |
| nonanal | NT | 1103 | 1101 | 1393 |  |  |  |  | 0.2 |  |  |  |
| 1-octen-3-yl-acetate | NT | 1112 | 1113 | 1386 | 0.8 | 2.7 | 0.2 | 2.1 | 0.3 |  | 1.0 |  |
| 3-octanol acetate | NT | 1126 | 1123 | 1339 |  | 0.4 |  | 0.1 |  |  | 0.3 |  |
| allo-ocimene | OM | 1130 | 1132 | 1383 | 0.3 | 0.4 | 0.3 | 0.9 | 0.5 | 0.5 | 1.4 | 0.3 |
| trans-pinocarveol | OM | 1140 | 1139 | 1677 | 0.4 | 0.1 |  |  | 0.4 | 0.1 |  |  |
| camphor | OM | 1148 | 1146 | 1507 | 3.4 | 3.0 | 2.4 | 1.1 | 2.4 | 3.4 | 0.3 | 3.6 |
| hexyl isobutyrate | NT | 1151 | 1152 | 1338 |  | 0.2 |  |  |  |  | 0.2 |  |
| (E)-2-nonenal | NT | 1162 | 1162^£^ | 1541 |  |  |  |  | 1.2 | 0.3 |  | 0.3 |
| pinocarvone | OM | 1166 | 1165 | 1573 | 0.3 | 0.1 |  |  |  |  |  |  |
| borneol | OM | 1169 | 1169 | 1693 | 4.9 | 4.6 | 4.9 | 2.7 | 4.3 | 6.6 | 2.4 | 6.7 |
| lavandulol | OM | 1186 | 1181 | 1678 |  | 0.6 |  |  |  | 1.0 |  |  |
| cis-pinocamphone | OM | 1175 | 1175 | 1553 |  |  |  | 0.2 |  |  |  |  |
| 4-terpineol | OM | 1178 | 1177 | 1602 | 1.4 | 0.9 | 1.1 | 5.8 | 1.8 | 3.6 | 7.7 | 1.8 |
| p-cymen-8-ol | OM | 1184 | 1183 | 1834 | 0.1 | 0.2 | 0.1 |  |  | 0.1 |  |  |
| cryptone | NT | 1187 | 1186 | 1668 |  |  | 0.3 | 0.2 | 2.4 | 0.3 |  | 0.3 |
| α-terpineol | OM | 1190 | 1189 | 1693 | 0.1 | 0.1 | 0.2 |  |  | 0.1 |  | 0.2 |
| hexyl butyrate | NT | 1192 | 1193 | 1414 | 0.3 | 0.9 | 0.4 | 0.5 | 0.7 | 0.2 | 1.0 | 0.3 |
| decanal | NT | 1205 | 1202 | 1500 | 0.4 | 0.2 | 0.1 | 0.7 | 0.3 | 0.3 |  | 0.1 |
| (E)-2-dodecene | NT | 1207 | 1205^£^ | 1262 | 0.1 |  |  |  |  |  |  |  |
| verbenone | OM | 1208 | 1205 | 1712 | 0.3 | 0.2 | 0.1 |  | 0.5 | 0.1 |  | 0.1 |
| cis-carveol | OM | 1230 | 1258 | 1852 |  |  |  |  | 0.1 |  |  |  |
| isobornyl formate | OM | 1234 | 1239 | 1596 | 0.5 | 0.5 | 0.4 | 0.2 | 0.8 | 0.7 | 0.1 | 0.5 |
| hexyl-2-methyl butyrate | NT | 1236 | 1236^£^ | 1427 | 0.1 |  | 0.1 |  | 0.1 |  |  |  |
| cuminaldehyde | OM | 1244 | 1242 | 1783 |  |  |  |  | 0.5 | 0.1 |  |  |
| hexyl-3-methylbutanoate | NT | 1243 | 1242^£^ | 1438 |  | 0.1 |  |  |  |  |  |  |
| carvone | OM | 1246 | 1243 | 1734 |  |  | 0.1 |  | 0.1 | 0.1 |  |  |
| linalyl acetate | OM | 1258 | 1257 | 1559 | 22.7 | 22.0 | 20.2 | 22.1 | 13.8 | 10.5 | 25.0 | 17.6 |
| bornyl acetate | OM | 1285 | 1289 | 1577 | 4.7 | 4.2 | 2.6 | 1.4 | 0.4 | 0.5 | 0.1 | 0.3 |
| lavandulyl acetate | OM | 1289 | 1290 | 1603 | 3.5 | 2.8 | 0.7 | 2.0 | 2.8 | 2.3 | 4.5 | 1.9 |
| hexyl tiglate | NT | 1331 | 1333 | 1602 |  |  | 0.1 | 0.2 |  |  |  |  |
| piperitenone | OM | 1343 | 1343 | 1937 |  |  |  |  | 0.2 | 0.1 |  |  |
| neryl acetate | OM | 1362 | 1362 | 1727 | 0.9 | 0.6 | 0.7 | 1.0 | 0.8 | 0.4 | 1.0 | 0.8 |
| geranyl acetate | OM | 1386 | 1381 | 1751 | 1.6 | 1.2 | 1.4 | 1.7 | 1.3 | 0.7 | 2.1 | 1.3 |
| tetradecane | NT | 1399 | 1400 | 1400 |  |  |  |  | 0.1 | 0.1 |  |  |
| dodecanal | NT | 1408 | 1409 | 1715 |  |  |  |  |  | 0.1 |  |  |
| α-cedrene | SH | 1409 | 1412 | 1578 |  |  |  |  |  |  |  | 0.1 |
| cis-α-bergamotene | SH | 1416 | 1413 | 1555 |  |  |  |  | 0.1 | 0.2 |  | 0.1 |
| β-caryophyllene | SH | 1418 | 1419 | 1593 | 7.8 | 2.3 | 11.7 | 9.1 | 8.8 | 9.9 | 6.2 | 13.9 |
| β-copaene | SH | 1429 | 1432 | 1483 | 0.1 |  |  |  | 0.1 |  | 0.2 | 0.2 |
| trans-α-bergamotene | SH | 1437 | 1435 | 1578 | 0.1 |  | 0.2 | 0.1 | 0.4 | 0.7 | 0.4 | 0.6 |
| aromadendrene | SH | 1441 | 1441 | 1607 | 0.1 |  | 0.1 |  | 0.2 | 0.2 | 0.1 | 0.2 |
| (Z)-β-farnesene | SH | 1443 | 1443 | 1646 |  |  |  |  | 1.3 |  |  |  |
| epi-β-santalene | SH | 1449 | 1447 | 1641 |  |  |  |  |  | 0.1 |  | 0.2 |
| (E)-geranyl acetone | AC | 1453 | 1455 | 1859 | 0.1 |  |  | 0.5 | 0.1 | 0.1 |  | 0.1 |
| α-humulene | SH | 1455 | 1455 | 1664 | 0.2 |  | 0.3 |  | 0.1 | 0.1 | 0.1 | 0.2 |
| (E)-β-farnesene | SH | 1460 | 1457 | 1661 | 1.0 | 1.6 | 0.5 | 0.2 | 5.0 | 1.7 | 1.4 | 1.6 |
| germacrene D | SH | 1480 | 1485 | 1699 | 1.0 | 0.3 | 2.2 | 1.3 | 1.9 | 1.8 | 1.3 | 3.3 |
| bicyclogermacrene | SH | 1494 | 1500 | 1769 |  |  |  |  | 0.1 | 0.1 |  | 0.1 |
| β-bisabolene | SH | 1509 | 1506 | 1729 |  |  |  |  | 0.1 |  |  |  |
| δ-cadinene | SH | 1510 | 1523 | 1749 | 0.1 |  |  |  | 0.1 |  |  |  |
| trans-γ-cadinene | SH | 1513 | 1514^£^ | 1767 | 0.1 | 0.6 | 0.6 | 0.2 | 0.1 |  |  |  |
| β-sesquiphellandrene | SH | 1525 | 1523 | 1771 |  |  |  |  |  | 0.1 |  |  |
| caryophyllene oxide | OS | 1582 | 1583 | 1986 | 1.9 | 0.1 | 0.5 | 0.1 | 0.3 | 0.5 | 0.1 | 0.9 |
| τ-cadinol | OS | 1642 | 1640 | 2171 |  | 0.2 | 0.1 |  |  |  |  |  |
| (E)-nerolidol acetate | OS | 1718 | 1717 |  |  |  |  |  |  |  |  | 0.1 |
| (Z)-lanceol | OS | 1763 | 1761 | 2449 |  |  |  |  |  |  |  | 0.2 |
| Class of compounds |  |  |  |  | **Susa** | | | **Stura** | | **Tanaro** | | |
|  |  |  |  |  | **H** | **M** | **L** | **H** | **L** | **H** | **M** | **L** |
| Monoterpene hydrocarbons (MH) | | | | | 9.5 | 11.8 | 8.9 | 19.9 | 11.3 | 16.9 | 22.4 | 12.6 |
| Oxygenated monoterpenes (OM) | | | | | 73.8 | 78.1 | 70.6 | 63.3 | 61.2 | 63.6 | 62.5 | 61.4 |
| Sesquiterpene hydrocarbons (SH) | | | | | 10.5 | 4.8 | 15.6 | 10.9 | 18.3 | 14.9 | 9.7 | 20.5 |
| Oxygenated sesquiterpenes (OS) | | | | | 1.9 | 0.3 | 0.6 | 0.1 | 0.3 | 0.5 | 0.1 | 1.2 |
| Non terpene derivatives (NT) | | | | | 2.2 | 5.1 | 1.2 | 4.6 | 6.1 | 1.5 | 3.2 | 1.1 |
| Apocarotenoid (AC) | | | | | 0.1 |  |  | 0.5 | 0.1 | 0.1 |  | 0.1 |

^£^ linear retention index from NIST web-site [https://webbook.nist.gov/chemistry/, 2018]
