# Supplementary material for: Latitude and Altitude Influence Secondary Metabolite Production in Peripheral Alpine Populations of the Mediterranean Species Lavandula angustifolia Mill
Source: Front Plant Sci. 2018 Jul 5;9:983. doi: 10.3389/fpls.2018.00983 (PMC6042283; doi:10.3389/fpls.2018.00983)
Supplement: Supplementary file 3 [file Data_Sheet_3.docx]

***Supplementary Material***

**Latitude and altitude influence secondary metabolite production in peripheral alpine populations of the Mediterranean species *Lavandula angustifolia* Mill.**

**Sonia Demasi, Matteo Caser, Michele Lonati, Pier Luigi Cioni, Luisa Pistelli, Basma Najar, Valentina Scariot^*^**

^*^**Correspondence**: Valentina Scariot, valentina.scariot@unito.it

**Supplementary Table 2.** Average abundance (%) of compounds identified in the EOs of the eight studied *L. angustifolia* populations, after two cultivation cycles under uniform growing conditions. The compounds are listed in their elution order on the DB-5 column. Compounds with relative percentages smaller or equal to 0.1% were excluded from the table and the analysis. *LRI_exp_*= experimental linear retention index relative to a series of alkanes; *LRI_lit_*= linear retention index from the literature on DB-5column (Adams, 2007); *LRI*=linear retention index on HP-Wax column. H=high altitude, M=medium altitude, L=low altitude, OM=oxygenated monoterpenes, MH=monoterpene hydrocarbons, SH=sesquiterpene hydrocarbons, OS=oxygenated sesquiterpenes, NT=non terpene derivatives, AC=apocarotenoid, vl= very low percentage

| Compound | Class | *LRI_exp_* | *LRI_lit_* | *LRI* | Susa | | | Stura | | Tanaro | | |
| --- | --- | --- | --- | --- | --- | --- | --- | --- | --- | --- | --- | --- |
|  |  |  |  |  | **H** | **M** | **L** | **H** | **L** | **H** | **M** | **L** |
| tricyclene | MH | 930 | 927 | 1003 |  | 0.1 | 0.2 |  | 0.1 | 0.1 | 0.1 | 0.1 |
| camphene | MH | 955 | 954 | 1058 | 0.4 | 0.6 | 0.8 | 0.1 | 0.3 | 0.3 | 0.1 | 0.2 |
| sabinene | MH | 976 | 975 | 1117 |  |  | 0.1 |  |  |  |  |  |
| 1-octen-3-one | NT | 980 | 980^£^ | 1302 |  | 0.6 |  | 0.3 | 0.2 |  |  |  |
| β-pinene | MH | 981 | 979 | 1081 | 0.6 |  | 0.3 |  |  | 0.1 |  |  |
| 1-octen-3-ol | NT | 978 | 979 | 1447 |  | 0.2 | 0.3 |  | 0.5 | 0.2 | 0.2 | 0.3 |
| 3-octanone | NT | 987 | 984 | 1262 | 1.0 | 1.2 | 0.3 | 0.5 | 0.6 | 0.5 | 0.5 | 0.3 |
| myrcene | MH | 990 | 991 | 1164 | 1.0 | 0.8 | 1.1 | 0.7 | 0.7 | 0.5 | 0.6 | 0.5 |
| butanoic acid butyl ester | NT | 993 | 995 | 1216 |  | 0.1 |  | 0.2 | 0.1 |  |  | 0.1 |
| 3-octanol | NT | 998 | 991 | 1398 | 0.2 | 0.2 | 0.1 |  | 0.1 | 0.1 | 0.2 |  |
| cis-dehydroxylinalool oxide | OM | 1009 | 1008 | 1210 | 0.1 | 0.1 | 0.1 |  | 0.1 | 0.1 |  |  |
| N-hexyl acetate | NT | 1013 | 1009 | 1276 | 0.8 | 0.3 | 0.1 | 0.5 | 0.1 |  |  |  |
| α-terpinene | MH | 1018 | 1017 | 1177 | 0.1 | 0.1 | 0.1 |  | 0.2 | 0.2 | 0.1 |  |
| o-cymene | MH | 1026 | 1026 | 1287 | 0.1 | 0.1 | 0.1 |  | 0.2 | 0.2 |  |  |
| p-cymene | MH | 1028 | 1025 | 1266 | 0.3 | 0.3 | 0.3 | 0.2 | 0.6 | 0.8 | 0.4 | 0.3 |
| limonene | MH | 1031 | 1029 | 1194 |  |  |  |  | 0.2 |  | 0.1 | 0.4 |
| 1,8-cineole | OM | 1034 | 1031 | 1209 | 2.6 | 4.2 | 9.2 | 1.4 | 1.5 | 3.2 | 1.1 | 0.2 |
| (Z)-β-ocimene | MH | 1041 | 1037 | 1242 | 0.6 | 0.5 | 1.1 | 0.9 | 0.3 | 0.2 | 0.6 | 0.6 |
| (E)-β-ocimene | MH | 1051 | 1050 | 1253 | 1.0 | 0.6 | 1.0 | 0.6 | 0.5 | 0.4 | 0.7 | 0.7 |
| γ-terpinene | MH | 1062 | 1060 | 1238 |  |  |  |  |  | 0.1 | 0.1 |  |
| trans-linalool oxide (furanoid) | OM | 1069 | 1073 | 1460 | 1.0 | 1.2 | 1.3 | 1.0 | 2.3 | 2.0 | 1.0 | 1.2 |
| cis-sabinene hydrate | OM | 1068 | 1070 | 1463 | 0.2 | 0.2 | 0.3 | 0.1 | 0.4 | 0.8 | 0.5 | 0.2 |
| cis-linalool oxide (furanoid) | OM | 1075 | 1087 | 1457 | 1.1 | 1.2 | 1.3 | 0.9 | 2.0 | 1.7 | 0.9 | 1.2 |
| camphenilone | OM | 1083 | 1082 | 1474 |  |  |  |  | 0.1 |  |  |  |
| 6,7-epoxymyrcene | OM | 1095 | 1093 | - | 0.6 | 0.6 | 0.4 | 0.6 | 0.5 | 0.5 | 0.3 | 0.2 |
| linalool | OM | 1099 | 1097 | 1550 | 19.4 | 14.3 | 25.1 | 15.9 | 26.0 | 23.7 | 24.1 | 35.0 |
| 1-octen-3-yl acetate | NT | 1112 | 1113 | 1386 | 3.5 | 3.8 | 2.4 | 4.6 | 1.8 | 0.5 | 1.1 | 0.3 |
| cis-p-menth-2-en-1-ol | OM | 1125 | 1122 | 1629 |  |  | 0.1 |  | 0.2 | 0.1 |  | 0.1 |
| 3-octanol acetate | NT | 1126 | 1123 | 1339 | 0.8 | 0.9 | 0.1 | 0.4 |  |  | 0.2 |  |
| α-campholenal | OM | 1130 | 1126 | 1495 |  |  |  |  | 0.1 | 0.1 |  |  |
| (Z)-myroxide | OM | 1137 | 1135 | 1521 | 0.4 | 0.2 | 0.2 | 0.3 | 0.2 | 0.2 | 0.1 |  |
| trans-pinocarveol | OM | 1140 | 1139 | 1677 | 0.2 | 0.2 |  |  |  | 0.1 |  |  |
| eucarvone | OM | 1146 | 1248^£^ | 1745 |  |  |  |  | 0.1 | 0.2 |  |  |
| camphor | OM | 1148 | 1146 | 1507 | 1.5 | 1.5 | 1.7 | 1.3 | 1.9 | 2.0 | 0.9 | 1.1 |
| trans-verbenol | OM | 1150 | 1145 | 1678 | 0.2 |  | 0.1 |  |  | 0.3 | 0.1 |  |
| hexyl isobutyrate | NT | 1151 | 1152 | 1338 |  | 0.4 | 0.1 | 0.2 | 0.2 |  |  |  |
| nerol oxide | OM | 1158 | 1158 | 1473 |  |  | 0.1 |  | 0.1 | 0.1 |  |  |
| pinocarvone | OM | 1166 | 1165 | 1573 | 0.4 | 0.2 | 0.2 | 0.1 | 0.1 | 0.2 | 0.1 |  |
| borneol | OM | 1169 | 1169 | 1693 | 3.6 | 4.4 | 4.8 | 2.7 | 5.9 | 6.7 | 3.6 | 4.6 |
| 4-terpinenol | OM | 1178 | 1177 | 1602 | 0.7 | 0.5 | 1.1 | 0.6 | 1.7 | 3.0 | 4.6 | 2.0 |
| cryptone | NT | 1187 | 1186 | 1668 |  | 1.0 | 0.9 | 0.2 | 1.3 | 0.7 | 0.4 | 1.0 |
| p-cymen-8-ol | OM | 1184 | 1183 | 1834 | 0.3 | 0.2 | 0.1 | 0.1 | 0.2 | 0.3 | 0.1 | 0.1 |
| α-terpineol | OM | 1190 | 1189 | 1693 | 4.3 | 2.9 | 4.6 | 3.0 | 3.1 | 2.1 | 3.2 | 3.0 |
| verbenone | OM | 1208 | 1205 | 1712 | 0.3 | 0.3 | 0.3 | 0.2 | 0.4 | 0.4 | 0.4 | 0.2 |
| trans-carveol | OM | 1221 | 1217 | 1838 | 0.9 | 0.6 | 0.2 | 0.6 | 0.6 | 0.5 | 0.4 | 0.2 |
| cis-p-mentha-1(7),8-dien-2-ol | OM | 1230 | 1231 | 1888 | 0.2 | 0.2 | 0.3 | 0.1 | 0.3 | 0.3 | 0.2 | 0.1 |
| isobornyl formate | OM | 1234 | 1239 | 1596 | 0.3 | 0.4 | 0.3 | 0.1 | 0.4 | 0.5 | 0.2 | 0.3 |
| nerol | OM | 1232 | 1230 | 1800 | 1.4 | 0.7 | 1.0 | 0.8 | 0.6 | 0.5 | 0.8 | 0.6 |
| 3-methyl-3hexen-1-yl butanoate | NT | 1236 | 1235 | - |  | 0.1 | 0.1 |  | 0.1 |  |  | 0.1 |
| cuminaldehyde | OM | 1244 | 1242 | 1783 |  | 0.5 | 0.5 | 0.2 | 0.8 | 0.4 | 0.2 | 0.6 |
| carvone | OM | 1246 | 1243 | 1734 |  | 0.3 | 0.2 |  | 0.4 | 0.2 | 0.1 | 0.3 |
| linalyl acetate | OM | 1258 | 1257 | 1559 | 20.6 | 16.0 | 17.0 | 26.3 | 12.5 | 11.9 | 18.4 | 13.6 |
| isopulegol acetate | OS | 1273 | 1278 | 1580 | 0.1 | 0.2 |  |  |  |  | 0.1 |  |
| isobornyl acetate | OM | 1287 | 1286 | 1568 | 1.6 | 1.7 | 1.6 | 1.1 | 0.5 | 0.5 | 0.5 | 0.2 |
| lavandulyl acetate | OM | 1289 | 1290 | 1603 | 4.2 | 6.7 | 1.2 | 5.2 | 3.6 | 2.5 | 5.7 | 3.0 |
| carvacrol | OM | 1301 | 1299 | 2209 | 0.1 |  |  | 0.2 | 0.3 | 0.6 | 0.8 | 0.1 |
| δ-elemene | SH | 1340 | 1338 | 1472 | 0.1 | 0.1 | 0.1 | 0.3 | 0.5 | 0.9 | 0.4 | 0.4 |
| α-terpinyl acetate | OM | 1352 | 1349 | 1694 | 0.1 |  |  |  |  |  |  |  |
| (Z)-8-hydroxylinalool | OM | 1360 | 1361^£^ | 2321 | 0.7 | 1.0 | 0.4 | 0.5 | 0.5 | 0.4 | 0.2 | 0.1 |
| neryl acetate | OM | 1362 | 1362 | 1727 | 1.9 | 1.2 | 1.5 | 1.9 | 1.2 | 1.0 | 1.6 | 1.2 |
| α-copaene | SH | 1376 | 1377 | 1483 |  |  |  |  |  |  |  | 0.1 |
| geranyl acetate | OM | 1386 | 1381 | 1751 | 3.8 | 2.4 | 3.2 | 3.8 | 2.6 | 2.2 | 3.6 | 2.5 |
| hexyl N-hexanoate | NT | 1388 | 1384 | 1606 |  |  |  |  |  |  |  | 0.1 |
| α-cedrene | SH | 1409 | 1412 | 1578 |  |  |  |  |  |  |  | 0.1 |
| β-caryophyllene | SH | 1418 | 1419 | 1593 | 1.1 | 0.8 | 1.7 | 2.2 | 1.7 | 2.4 | 2.7 | 3.7 |
| trans-𝛂-bergamotene | SH | 1437 | 1435 | 1578 |  |  |  | 0.1 | 0.2 | 0.2 | 0.2 | 0.3 |
| aromadendrene | SH | 1441 | 1441 | 1607 |  | 0.2 |  | 0.1 | 0.1 | 0.2 | 0.2 | 0.2 |
| epi-β-santalene | SH | 1449 | 1447 | 1640 |  |  |  |  | 0.1 | 0.1 | 0.1 | 0.1 |
| (E)-β-farnesene | SH | 1460 | 1457 | 1661 | 0.3 | 0.2 | 0.2 | 1.1 | 0.2 | 0.2 | 0.9 | 0.9 |
| germacrene D | SH | 1481 | 1485 | 1699 | 0.1 |  | 0.1 | 0.3 | 0.1 | 0.1 | 0.4 | 0.7 |
| γ-curcumene | SH | 1484 | 1483 | 1695 |  |  |  |  |  | 0.1 | 0.2 | 0.2 |
| trans-γ-cadinene | SH | 1513 | 1514^£^ | 1767 |  | 1.4 | 0.3 | 0.2 | 0.1 |  |  |  |
| (Z)-γ-bisabolene | SH | 1515 | 1507 | 1770 | 0.7 | 0.5 | 0.5 | 1.5 | 2.0 | 3.1 | 1.8 | 1.5 |
| (E)-γ-bisabolene | SH | 1535 | 1531 | 1763 | 0.2 | 0.1 | 0.2 | 0.6 | 0.8 | 1.3 | 0.9 | 0.8 |
| cis-sesquisabinene hydrate | OS | 1545 | 1544 | 2078 |  | 0.1 |  | 0.2 | 0.3 | 0.5 | 0.3 | 0.2 |
| elemol | OS | 1553 | 1550 | 2086 | 0.8 | 0.5 | 0.4 | 0.7 | 0.7 | 0.9 | 0.5 | 0.6 |
| germacrene B | SH | 1556 | 1561 | 1820 |  | 0.1 |  | 0.1 | 0.2 | 0.3 | 0.2 | 0.3 |
| spathulenol | OS | 1581 | 1578 | 2129 |  | 0.2 |  | 0.2 | 0.3 | 0.5 | 0.1 | 0.3 |
| caryophyllene oxide | OS | 1582 | 1583 | 1986 | 9.3 | 5.5 | 4.7 | 9.0 | 7.0 | 7.6 | 5.4 | 7.1 |
| thujapsan-2-α-ol | OS | 1589 | 1587 | - |  |  | 0.1 | 0.4 | 1.6 | 2.4 | 1.6 | 1.1 |
| humulene epoxide II | OS | 1607 | 1608 | 2068 | 0.2 | 0.2 | 0.1 | 0.2 | 0.2 | 0.3 | 0.2 | 0.2 |
| 1,10-di-epi-cubenol | OS | 1614 | 1613^£^ | 2056 | 0.1 | 0.8 | 0.2 | 0.2 | 0.2 | 0.2 | 0.2 | 0.2 |
| α-acorenol | OS | 1633 | 1633 | 2163 |  | 0.2 |  |  | 0.1 | 0.1 |  |  |
| β-acorenol | OS | 1636 | 1637 | - |  |  |  |  | 0.1 | 0.2 | 0.2 | 0.1 |
| cis-cadin-4-en-7-ol | OS | 1637 | 1637 | 2114 |  |  |  |  |  |  | 0.1 |  |
| β-caryophylla-4(14),8(15)-dien-5-ol | OS | 1639 | 1641 | 2290 | 0.2 |  |  | 0.1 | 0.2 | 0.3 | 0.2 | 0.2 |
| τ-cadinol | OS | 1642 | 1640 | 2171 | 0.6 | 7.6 | 2.0 | 1.0 | 0.5 | 0.1 | 0.3 | 0.1 |
| α-cadinol | OS | 1655 | 1654 | 2222 | 0.1 |  |  |  | 0.2 | 0.4 | 0.4 | 0.4 |
| neo-intermediol | OS | 1660 | 1660 | - |  |  |  | 0.3 | 0.3 | 0.3 | 0.2 | 0.1 |
| (Z)-α-santalol | OS | 1660 | 1675 | 2306 | 1.5 | 0.5 | 0.4 | 1.1 | 0.8 | 0.7 | 1.3 | 0.9 |
| (Z)-nerolidol acetate | OS | 1668 | 1678 | - |  |  |  |  |  |  | 0.3 | 0.2 |
| 14-hydroxy-9-epi-(E)-caryophyllene | OS | 1672 | 1670 | 2354 | 0.3 | 0.1 |  | 0.3 | 0.2 | 0.2 |  | 0.2 |
| elemol acetate | OS | 1675 | 1681 | 2026 |  |  |  |  | 0.6 | 0.8 |  | 0.6 |
| cis-14-muurol-5-en-4-one | OS | 1684 | 1689 | - | 0.3 | 1.6 | 0.5 | 0.5 | 0.2 |  | 0.2 | 0.4 |
| acorenone B | OS | 1698 | 1698 | - |  |  |  |  |  |  | 0.1 |  |
| 14-hydroxy-α-humulene | OS | 1714 | 1714 | 2475 |  |  |  |  |  | 0.2 | 0.2 | 0.1 |
| curcuphenol | OS | 1720 | 1718 | - |  | 0.2 |  |  |  | 0.1 |  |  |
| isolongifolol | OS | 1726 | 1730 | - |  | 0.1 |  |  |  |  |  |  |
| cedr-8(15)-en-9-α-ol acetate | OS | 1743 | 1743 | - |  | 0.4 | 0.1 |  |  |  |  |  |
| cyclocolorenone | OS | 1758 | 1761 | 2354 | 0.1 | 1.3 | 0.3 | 0.2 |  |  |  |  |
| benzyl benzoate | NT | 1760 | 1760 | 2633 | 0.1 | 0.2 |  |  | 0.2 |  |  |  |
| (Z)-α-santalol acetate | OS | 1763 | 1779 | - | 0.1 | 0.1 |  |  | 0.1 | 0.1 |  |  |
| β-chenopodiol | OS | 1810 | 1812 | - |  | 0.1 |  |  |  |  |  |  |
| hexahydrofarnesylacetone | AC | 1845 | 1846^£^ | 2114 | 0.2 | 0.3 | 0.2 | 0.4 | 0.4 | 0.6 | 0.3 | 0.1 |
| Yield in EO (%) |  |  |  |  | vl^*^ | 0.35 | 0.69 | 0.28 | 0.15 | 0.18 | vl | 0.37 |
| Class of compounds | | | | | **Susa** | | | **Stura** | | **Tanaro** | | |
|  |  |  |  |  | **H** | **M** | **L** | **H** | **L** | **H** | **M** | **L** |
| Oxygenated monoterpenes (OM) | | | | | 72.5 | 63.7 | 77.0 | 68.8 | 71.1 | 69.3 | 73.7 | 73.3 |
| Monoterpene hydrocarbons (MH) | | | | | 4.0 | 3.2 | 5.1 | 2.4 | 3.0 | 2.9 | 2.7 | 2.6 |
| Sesquiterpene hydrocarbons (SH) | | | | | 2.6 | 3.5 | 3.5 | 6.6 | 6.1 | 8.8 | 8.1 | 9.8 |
| Oxygenated sesquiterpenes (OS) | | | | | 13.8 | 19.7 | 9.0 | 14.5 | 13.5 | 15.7 | 11.9 | 11.8 |
| Non terpene derivatives (NT) | | | | | 6.3 | 8.9 | 5.1 | 6.8 | 5.2 | 2.0 | 2.7 | 2.1 |
| Apocarotenoid (AC) | | | | | 0.2 | 0.3 | 0.1 | 0.4 | 0.4 | 0.6 | 0.3 | 0.1 |

^£^ linear retention index from NIST web-site [https://webbook.nist.gov/chemistry/, 2018]
